# Syndrome of Irreversible Lithium-Effectuated NeuroToxicity: SILENT, but not innocent

**DOI:** 10.1192/j.eurpsy.2023.1469

**Published:** 2023-07-19

**Authors:** M. S. Bicho, J. M. Coelho, H. J. Fontes

**Affiliations:** Psychiatry, Hospital do Divino Espírito Santo de Ponta Delgada, EPER, Ponta Delgada, Portugal

## Abstract

**Introduction:**

Lithium is one of the main drugs used in Bipolar Affective Disorder. However, it has a narrow therapeutic window, which requires close monitoring and progressive dose adjustment, according to serum levels, clinical response and the appearance of side effects. The term ‘SILENT’ explains descriptively persistent neurological sequelae related to lithium salt intoxication when symptoms persist for more than 2 months after stopping treatment.

SILENT Syndrome is more common in females, at ages ranging from 21 to 77 years and is characterized mainly by avermian-type cerebellar disorder, persistent extrapyramidal syndrome, brainstem dysfunction and dementia of varying severity. It can also result in apraxia of the body, changes in the coordination and balance, dysarthria, as well as intentional and kinetic cerebellar tremor, involuntary movements of orofacial dyskinesias or resting tremor.

**Objectives:**

The authors intend to review the relevant and current literature in order to extend the knowledge about this condition and find the best conducts for clinical practice.

**Methods:**

Non-systematic literature review.

**Results:**

Complications from the use of lithium known in the medical literature include mainly nephrotoxicity, endocrine alterations and neurotoxicity.

The neurotoxic effects of lithium usually occur at high serum concentrations. However, they can also occur with lithium in the therapeutic range, and memory, attention and ataxia impairment may be some of the permanent sequelae.

The etiopathogenesis is unclear, but demyelination has been detected in multiple brain regions, mainly in the cerebellum. The mechanism of lithium-induced cerebellar injury is believed to be mediated by the entry of calcium into the cells of this organ.

The main factors that predispose to greater side effects and risk of toxicity are patients with decreased renal function, advanced age, use of diuretics, dementia, pregnancy, low sodium intake and physical illness with vomiting and/or diarrhea.

**Conclusions:**

Lithium is a drug used mostly in affective disorders and given the narrow therapeutic window, it requires close monitoring in order to avoid side effects that can be permanent. In this way, it is important to review the factors that increase the lithium toxicity and make recommendations about it.

**Disclosure of Interest:**

None Declared

